# Changes of Location

**Published:** 1916-04

**Authors:** 


### Changes of Location.

Martin H. Smith Co., 105 Chambers Street, New York, announce their removal to 150-156 Lafayette Street.

Dr. E. O. Arnold of Rockport has moved to Corpus Christi to resume his practice.

Dr. G. A. Foote, formerly of Byers, but who has recently been traveling with his wife for the benefit of her health, has located at Alpine, where the doctor will practice his profession.

Dr. and Mrs. Owen of Arlington have moved to Dallas.

Dr. H. E. Stolp, who for the past two years has been in Denver, Colo., has moved to Bowie to make his future home.

Dr. Chas. B. Townes of Sanger has moved to Tahoka, where the doctor will practice medicine and surgery.

Dr. J. A. Odom and family of Rodgers have moved to Childress to make their future home. Dr. Odom has been in charge of the Rodgers Sanitarium for a number of years, and for the past two years has been specializing in ear, eye, nose and throat diseases.

Dr. H. Wichmann of Mobeetie has gone to El Paso, Texas, where he will probably proceed to Mexico City to take up the duties of an army surgeon.

Dr. J. H. Price of Knox has moved to Lamesa, where he will be engaged in the building of a sanitarium.

Dr. E. M. Parrish of Gainesville has gone to Dallas, where he will make his future home.

Dr. O. E. Looney of Paducah has located at Swearingen.

Dr. H. K. Cansler has moved from Hillsboro to Arlington, where he will engage in the practice of his profession.

Dr. J. F. Baugh of Poyner has recently located in Chandler.

